# Assessing the impact of university students’ involvement in the first year of Nurture-U: a national student wellbeing research project

**DOI:** 10.1186/s40900-023-00478-7

**Published:** 2023-10-17

**Authors:** Jemima Dooley, Amina Ghezal, Thomas Gilpin, Husna Hassan Basri, Katy Humberstone, Amber Lahdelma, Pranati Misurya, Ellen Marshall, Ed Watkins

**Affiliations:** 1 Sir Henry Wellcome Centre for Mood Disorders Research, University of Exeter, Devon, EX4 4QG UK; 2 Department of Politics, University of Exeter, Penryn, Cornwall, TR10 9FE UK; 3 Coronary Research Group, University Hospital Southampton, Southampton, SO16 6YD UK; 4 Faculty of Medicine, University of Southampton, Southampton, SO17 1BJ UK; 5 Department of Psychology, University of Exeter, Devon, EX44QG UK; 6 Department of Languages, Cultures and Visual Studies, University of Exeter, Devon, EX4 4QH UK; 7 Department of Languages, Cultures and Linguistics, University of Southampton, Hampshire, SO17 1BF UK; 8 Combined Honours Department, Peter Chalk, University of Exeter, Devon, EX4 4QD UK; 9 School of Psychology, Newcastle University, Newcastle upon Tyne, NE1 7RU UK

**Keywords:** Mental health, Co-design, Public involvement, Students, Co-production, Public engagement, Wellbeing

## Abstract

**Background:**

Students experience lower levels of wellbeing than the general, age-matched population. A whole-university approach to mental health is encouraged, which must work for individuals from all backgrounds and experiences. Student input is vital in researching and designing these solutions. Nurture-U is a national, large-scale research project exploring better ways to support student wellbeing, with a Student Advisory Group (SAG) that feeds into project decision making. With the first year of the project now completed, we now critically review the processes and effectiveness of the SAG and how well the project is engaging and working with students.

**Methods:**

Assessment of the SAG’s impact on the project, the student advisors, and the researchers was undertaken through a content analysis of team meetings and collection of advisor and researcher feedback using the Patient Engagement Quality Guidance Tool.

**Results:**

142 students worked on different tasks in the first year of the Nurture-U project. The SAG was involved in the project branding and marketing, and in the development and co-design of interventions and tools. They reported a positive experience, with involvement boosting confidence. They felt valued but reported not always knowing whether their input was implemented in final decisions. They also recommended different methods of providing feedback. Researchers found student input beneficial to communicate the viewpoint of a different generation and increase the relevance of the study, but also suggested improvements for communication between the research team and the student group.

**Conclusions:**

This critical reflection of the SAG’s public advisor role in this large-scale research project was important in highlighting what worked well and areas to improve. As the project unfolds, we aim to adapt our methods of student input, increase the transparency of decision-making processes, and in turn increase student-led decision making within the project.

## Abstract (plain language summary)

University students face many challenges to their wellbeing, including academic stress, frequent changes of location, distancing of support systems, and new social and cultural surroundings. The Nurture-U research project is looking at how universities can support student wellbeing. It is supported by a Student Advisory Group (SAG) that is helping us design the research. The aim of this paper is to reflect on the input of the SAG in the first year of the 4-year Nurture-U project. 142 students worked on different tasks in the first year of the Nurture-U project, including marketing and designing therapeutic apps. The SAG described feeling valued, gaining confidence and an understanding of mental health and research. Researchers felt the SAG improved Nurture-U, making it more relevant to students. However, all were concerned that final decisions were researcher-led, not student-led. There was also a lack of diversity, with most of the SAG being female. The SAG advised different ways for researchers and students to work together. This included having different ways for student advisors to voice their opinions, for example in smaller or face-to-face groups, or on online boards. Also, researchers could engage with the SAG differently, through newsletters or videos, to make it clearer how final decisions are made. This formal reflection activity has helped the Nurture-U team consider ways to improve working with the SAG for the rest of the project, which we hope will ensure that student voices guide how the project unfolds.

## Background

Including young people in mental health research is vital to reduce power imbalances and mitigate inequalities, but little has been written about specifically how this is done and its impact on those involved [1]. Research into student wellbeing is in its infancy, with the majority of work being undertaken in the last decade [2]. Organisations such as Student Minds (https://www.studentminds.org.uk/) and the SMaRteN (https://www.smarten.org.uk/) network have shown how students themselves should be setting agendas for future research, play a role in how it is conducted, and have a say in how results are used and implemented [3]. The Nurture-U project is a £4 million national research study, linked with SMaRten, that aims to explore a whole-university approach to student wellbeing within 6 universities: Cardiff, Exeter, King’s College London, Oxford, Newcastle, and Southampton.

Rates of low mood, high anxiety and stress are higher in students than the general population [4]. Most university students are under 25 years old, an age vulnerable to the development of mental illness [5]. Contributing factors are typically a lack of social support, frequent transitions between home and university, the cost of living, and high academic stress [6]. There is also a recognised gap between students with different experiences and backgrounds. For example, autistic students, those who identify as LGBTQ+, and those who experienced trauma in childhood have increased risk of developing poor mental health at university [7]. Hence, there is a need both to improve the general wellbeing of students and to tailor care to be more personalised. Including students themselves in these conversations can help address these complex issues [1]. The SMaRteN network has laid important groundwork for this by conducting a national survey on the areas which students identify as important topics for research [3].

Nurture-U began in October 2021, with completion planned for July 2025. It is led by the University of Exeter, partnered with the Universities of Cardiff, Kings College London, Newcastle, Oxford, and Southampton (see www.nurtureuniversity.co.uk for more details). This paper reports on the impact of the Student Advisory Group (SAG) on the first year of the project, co-written with members of the SAG (authors AG, TG, HH, KH, AL, and PM). While the importance of public involvement in research is well established, involvement in mental health research has its own challenges. Stigma, burden, and differing expectations as to research scope can all constitute barriers to participation [8]. Indeed, research into co-production of young people’s mental health services highlights the need to allow young people with mental health needs to discuss their own experiences within the collaborative space [9]. Additionally, it is important to critically explore issues such as the sharing of power in the decision making process and the diversity of public contributors, so as to ensure involvement is not tokenistic [10]. The aim of this paper is to consider these issues while outlining how and where students impacted the first year of the Nurture-U project, and assessing the personal impact of student involvement in the project on the student advisors and the research team.

## Methodology

### Project outline

The Nurture-U research project consists of 5 workstreams: a biannual wellbeing survey, trialling an electronic Wellbeing Toolkit, developing and evaluating a mental health literacy course, the Compassionate Campus project, and three research trials of different interventions (see Table 1 for overview). While there was student input in the project conception and planning the grant application, a separate Nurture-U SAG was set up in the initial months of the project to aid the design, conduct, analysis and dissemination of each workstream.

**Table 1:** Summary of project and student input

Area of the project	Details	Student input	Method of student involvement	Specific advice and suggestions	Impact on the project
Student engagement	Project needs to engage as many different types of students as possible to produce valid and relevant results	Branding	Discussed over four group meetings. Student submitted names, logos and taglines. Three voting rounds	Name: short, encompass project aims, inclusive to all types of students. Put forward selection of ideas, final vote was between ‘Nurture’ and ‘Thrive’. Nurture chosen to be more indicative of support. Inclusion of ‘U’ to represent ‘you’ and ‘university Logo: promote inclusivity, community, safety, support, independence, and happiness. Green and/or blue, blue is overused in both universities and the NHS. Rounded images relating to nature or human togetherness (e.g. hands and arms). Traditional mental health images such as brains are too clinical. The idea of the N and U in Nurture-U as an infinity symbol was put forward and voted as the best. Infinity symbol was found to already exist, so the N and U were separated. The plant was added by one of the student designers and the group reported that it represented growth and support Tagline: Student advisors favoured ‘Student wellbeing starts with U’, a student designed tagline, with ‘Finding better ways to support students’ voted as second best	Name: chosen by group and research team Logo: student design taken to graduate graphic designer for final product Tagline: ‘Finding better ways to support students’ was deemed by the research team to better represent the project aims
		Website and social media	Planning and design discussed over four group meetings. Teams channel and canva design for ongoing student involvement in content	Mood board created by the research team based on the discussions in logo development. Advisors voted on beige, green and yellow aesthetic, seen as bright and distinctive but also soft and calming Website created by the research team. Student advisors fed back on accessibility and readability. The ‘Mental Health Advice’ page was reported as too overwhelming and hence unhelpful. This feedback led to extensive re-design by the research team Students provided self-filmed video clips using a script written by the research team that is placed on the home page of the website A social media strategy was developed: Instagram seen as the most relevant to both the project and students. Tik Tok most likely to reach the most students, but there was recognition of the difficulty of maintaining relevance. Facebook and Twitter were seen as necessary, but likely to have less reach Advised on tag and bio, how to manage content, and the best times of day to post Importance of Instagram ‘giving something back’ to followers, rather than being just a promotional tool. Development of psychoeducational posts	Setting of brand colours, tone and images to use online, which were mirrored in on-campus promotional materials Vast improvement of the study website Development of promotional video Social media strategy and content design and contribution
Survey	Wellbeing survey takes place twice a year over three years	Survey promotion	Planned and discussed over 3 meetings Student run stalls on campus Student presentations in lectures.	Suggestions and options based both on advisor knowledge of student life and previous experience running on-campus promotion. Included: best timings, locations, and most appealing merchandise and ‘freebies’. Food and hot drinks seen as most appealing Advised regular stalls to increase knowledge and presence of the brand Identified the strategy of going into lectures and promoting the survey to a captive audience	Events and merchandise suggestions constrained by budget, but only student generated ideas were used Freshers week stalls (with free stress balls) raised over 300 instagram followers Regular, student run on-campus stalls with free food and drinks was by far the most successful method of survey recruitment Student presentations worked well in lectures, where research teams were able to make connections with lecturers Other recruitment methods, including social media adverts and mass student emails were not designed by the student group
Nurture-U Wellbeing Toolkit	The Nurture-U Wellbeing Toolkit is a programme called i-Spero: a web-based app that allows users to answer questions regular intervals so they can track their wellbeing over time. Users receive messages based on their answers to the questionnaires. There are also ‘Wellbeing Plans’, for example different services, apps, webpages or lifestyle changes that might help users	Co-design of Wellbeing Toolkit	Planned and designed over 6 meetings Two week trial of prototype with feedback	Researchers explained what needed to be included for research purposes, as well as what could not be changed within the formatting of the software, students were asked to co-design all other aspects Fourteen standardised wellbeing measures were built into the Toolkit, 11 further questionnaires were added on student advice. Students advised keeping questions to a minimum and text as brief as possible, to not overwhelm the user I-Spero is used within health services, and the language was formal and clinical. Students advised changes (for example the use of emojis) and added positive and motivational messaging, rather than just ‘alerts’ when wellbeing was low Highlighted the importance of the accessibility of the plans and making them relevant to diverse cultures and the disabled population Student testing of the demo version highlighted many bugs and usability issues	All student generated questionnaires were included in final product Messaging transformed, with aim to be supportive and positive where possible Wellbeing plans were designed to match the services provided by the university alongside evidence-based therapies, so students had less input on content. But on advice, the text was kept brief and to the point. Also more plans were added to increase inclusivity Student testing meant issues were addressed and content refined before release Some student suggestions were not included. For example, students wanted more personalisation and motivational tools, which was not possible in the current format of the software. There was also enthusiasm for adding a social aspect to the Toolkit, but the research team were concerned about data management and a lack of capacity to monitor adjacent forums or online communities
Compassionate Campus	Finding ways for universities to move away from ‘consumer-led’ student experience, to increasing kindness on campus	Development of social prescribing directory	Planned and designed over 5 whole-group meetings and further student-led meetings	Student interns led on the design and creating of online social prescribing directory Students led on finding and contacting groups and organisations for inclusion in the directory Students led on website design	Student-led design of directory to be tested by personal tutors.
Research trials	Trial 1: Testing self-guided rumination app ‘Minddistrict’, to see if it can prevent the onset of depression.	Feedback and testing content of app Deciding on terminology (And below)	One initial meeting. Two rounds of testing and feedback One group discussion and Microsoft form vote on terminology	Provided detailed feedback on the app and the research team changed the content based on this. Positive feedback on existing language and formatting, reporting that it was accessible and balanced. Detailed suggestions for changes that would increase clarity and inclusivity Students were asked whether ‘self-help’ was an acceptable term for these sorts of apps. Students voted that ‘self-guided’ was preferable	Student input increased the diversity of the student stories within the app Images and avatars chosen based on student preferences ‘Self-guided’ is now the term used by researchers in describing non-therapist guided interventions
	Trial 2: Comparing self versus therapist-guided Silvercloud therapy app	Testing RedCap software for trial participation (relevant for all trials)	Students provided cribsheets to test different RedCap pathways General feedback given via Microsoft forms	Changes in wording and language used Spotting inconsistencies in the information given, for example how long data was stored. Highlighting where error messages occurred Suggestions to improve usability, for example colours	Changes implemented by the trials unit
	Trial 3: Testing resilience programme for students	(as above)	(as above)	(as above)	(as above)

### Recruitment and conduct of the Nurture-U Student Advisory Group

We began recruitment to the SAG in November 2021 using the Universities’ student communications teams, networks, societies, and groups. The group was further promoted in April and June 2022. Recruitment has continued through word of mouth and social media presence.

The only inclusion criteria for participation in the SAG was that members had to be current under- or postgraduate students. There was no selection process; students applied to join the SAG, were added to a mailing list, and joined meetings when suited them. Lived experience of mental health challenges or using wellbeing services was not necessary. All members were made aware they could talk to the research team if they were experiencing any distress, and there was a risk protocol in place if any concerns were raised.

Students were inducted to the SAG through individual or group meetings, or virtually through email and a recorded presentation. Prior to joining, participants were sent Terms of Reference for group participation, information on payment, and an information sheet and consent form. Students are paid £10 an hour for their involvement (in line with minimum wage for over 23s in the UK).

Termly meetings are held virtually on Microsoft Teams, involving a project update and breakout rooms for discussion, finishing with group feedback. Meeting are led by the Student Engagement Officer (author JD). Students also lead and contribute through sub-groups with more specific foci, e.g., social media or intervention development. This might involve further Teams or hybrid meetings, providing written feedback, voting on Microsoft Forms or group work on Teams channels. After each meeting, summaries are sent to the whole group. Regular updates are also provided by email.

### ‘Patient Engagement Quality Guidance Tool’ (PEQG)

This tool was chosen as a framework to assess the quality of engagement as it is the most widely used tool for assessment of ongoing engagement of ongoing projects [11, 12]. It has seven criteria for assessment: shared purpose, respect and accessibility, representativeness, roles and responsibilities, capacity, transparency in communication and documentation, and continuity and sustainability.

### Evaluating impact

**Fig. 1:**
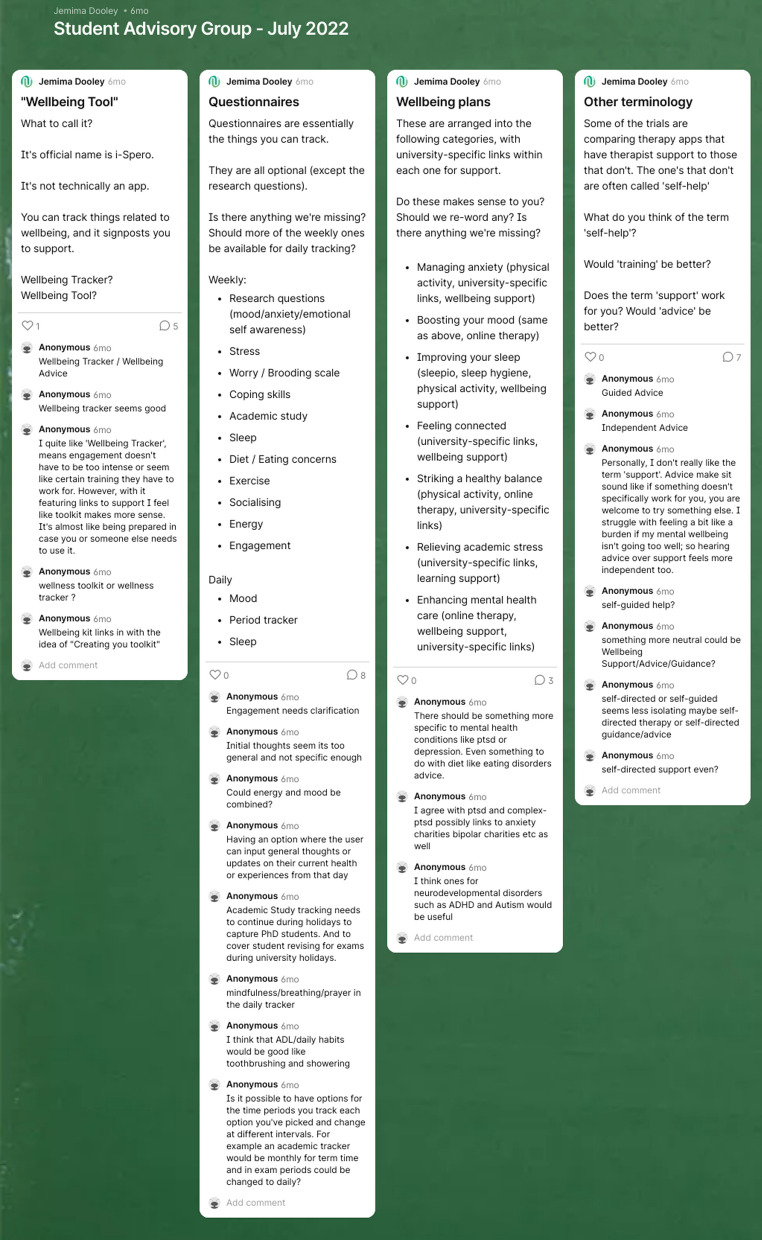
Example SAG Padlet

The impact of the SAG was explored in 3 areas:

1. The research project: impact was identified through content analysis of meeting summaries, Padlets (online ‘walls’ where you can post content, see Fig. 1 for an example), and Microsoft Forms used with the group. All data was transferred to NVivo and coded first according to content within each research activity. Codes were then categorised into themes summarising how these activities impacted the project.
2. The student advisors: impact was measured using a Microsoft Forms questionnaire. Questions were created following the seven categories outlined in the PEQG (see Table 2 for details). The questionnaire was sent to all participants in December 2022.
3. The research team: impact was identified through a Microsoft Form questionnaire with modified versions of the questions in Table 2 to make them applicable to the research team.

**Table 2:** Student PEQG questionnaire with Multiple Choice Results

Question	Multiple choice result
In your view, what is the purpose of the Nurture-U research project?	N/A free text
Do you think the purpose of the Nurture-U project has been communicated clearly?	Yes: 17 No: 0 Partly: 4 Don’t know: 0
Do you feel respected by the Nurture-U team and other students in the group?	Sometimes: 0 Yes: 21 No: 0 Don’t know: 0
If you can, please elaborate on what it is that makes you feel respected, or not respected, in the group	N/A free text
Are there things that we can change that would make you feel more respected or listened to in the group?	N/A free text
Could you please elaborate on what happened if you have every felt unable to talk about anything with the group and/or the research team? And what could have been done differently?	
Have there been times you have felt unable to voice your opinion about something?	Yes: 0 No: 18 Kind of: 3
Do you think the student group is sufficiently diverse/representative?	Yes: 11 No: 3 Not sure/Couldn’t say: 6 (1 missing)
How could we increase the diversity/representativeness of the group?	N/A free text
Have you felt clear about your role in the group? E.g. what is expected of you, how to contribute, how to ask questions?	Yes: 18 No: 0 Not sure: Partly: 3
If yes, what helped make it clear? And if no, what can we change to make it clearer in the future?	N/A free text
Would any training or further information have improved your experience at any point?	Yes: 6 No: 6 Not sure: 8 (1 missing)
If yes, what sort of thing could this be? If no, what helped you feel suitably capable for your role?	N/A free text
Is the information we send out before/after meetings useful?	Yes: 19 No: 0 Could be improved: 2 Not sure: 0
How can we improve this communication? E.g. shorter, longer, in video/audio form	N/A free text
Do you feel like you’re part of the Nurture-U team?	Yes: 18 It’s a bit ‘us and them’: 3 No: 0 Not sure: 0
If yes, what makes you feel part of the team? If no, how can we improve the relationship between the Nurture-U researchers and the Student Advisory Group?	N/A free text
Has being part of the group had any positive impact on you? Please add details	N/A free text
Has being part of the group had any negative impact on you? Please add details	N/A free text

For both the student and the research team questionnaires, quantitative data was collected within Microsoft Forms. Free text data were exported and collated according to content to allow categories for description below.

## Results

### Involvement and demographics

142 students were involved at different points in the opening year of the project (December 2021–December 2022). Sixteen meetings were held, with a median attendance of 23. Seventeen polls relating to decisions about the project were conducted between meetings, with between 7 and 38 responses. Twenty-six students were involved in social media and on-campus campaigns. The cumulative cost of student involvement was £8270.

The group consists of students from 5 of the 6 participating universities, but 89% were enrolled at the University of Exeter where the Student Engagement Lead (JD) is based. Demographic data has been collected for 96 students: 79% were female, 48% were Asian or Asian British, 42% White, 62% were international students, 23% were LGBTQ+, and 19% reported a disability.

### Impact on the research project

Specific details of the impact of the SAG are in Table 1. Below is a summary of the broad areas in which the advisors benefited the project, obtained from the analysis of meeting summaries and Padlets.

#### Inclusivity

A key contribution of the SAG was ensuring inclusivity. This involved discussions around promoting the positive message of wellbeing without deterring groups who do not already engage in these narratives or placing too much pressure on individuals to achieve an elusive state of happiness (*“The name needs to be gender neutral. not too ‘flowery’. not too goal oriented’”* Name and Logo Meeting 1). This was reflected in the neutral and simple branding of the study (see www.instagram.com/nurture_uni for examples), as well as the student-designed engagement campaign which emphasised the importance of cross-campus promotion (*“not all students are based in the same locations”* Marketing Meeting 2). The inclusion of free food and drinks was the most successful survey recruitment strategy and increased access to a wide demographic of the student population, rather than just those who may respond to email or social media links. The co-designed questionnaires and wellbeing plans in the Wellbeing Toolkit also ensured that these reflected the experiences of neurodivergent students, with a disability, or from different cultural backgrounds (*“We need to think about cultural factors and include ways to personalise to benefit people from different backgrounds”* Toolkit Meeting 1). This was also the case in the development of the Minddistrict app for the Rumination Trial (see Table 1 for details).

#### Accessibility

The SAG provided constant reminders to the research team about the need for all materials to be accessible (*“Students don’t want to be lost in a sea of information”* Toolkit Meeting 2). This was especially beneficial due to the scale of the project. The website was transformed after student feedback reported too much text, too little space between images and text, colours not being suitable for those who are colour blind and too little distinction between the pages. Results were similar in relation to the Wellbeing Toolkit, questionnaires were significantly shortened and restructured to avoid overloading. Student testing of the Toolkit, Minddistrict app, and RedCap software maximised usability and accessibility of these student-facing aspects of the study.

#### Relevance

Advisors specified what diverse student bodies may experience at different times of year, term, and day and how to target content and advertising accordingly. The marketing plan for the survey was co-designed with the SAG, who emphasised the complexity of student schedules and the importance of timely intervention to increase engagement (e.g. *“Keep campus appearances regular, not just once—people will forget”* Marketing Meeting 2). This resulted in promotion in lectures and stalls in the busiest areas. The addition of 11 SAG-designed questionnaires in the Wellbeing Toolkit means that it has been designed to capture the most applicable aspects of student wellbeing. In the development of the social media strategy and content, students recommended provision of psychoeducation and research results so that the study ‘gives something back’ to those who follow. This has entailed student involvement in dissemination of preliminary findings even at this early stage of the project.

### Impact on student advisors

Twenty-one advisors filled out the feedback questionnaire, out of the 142 who had participated over the opening 12 months of the project (5%). Student responses to multiple choice questions can be found in Table 2, and responses to the free text questions are quoted below. SAG co-authors (AG, TG, HH, KH, AL, and PM) also contributed their own experiences to this section by writing their own detailed feedback. Quotations are labelled as ‘respondent’ (R) or author (A).

#### Overall impact

Being a SAG Member was reported to be *“interesting”* (respondent (R)18), *“diverse”* (R3) and *“enriching”* (A). When asked to rate their experience out of 5, the median score was 5 (mean 4.5).

A key benefit was a sense of belonging (*“feeling part of a bigger community of students”* (R5)). It was reported as both exciting and rewarding to see a group-led initiative engage a wide student body, with the promise of supporting students longer-term. There is a strong sense of pride, knowing the SAG are doing something proactive by participating in a project which is so important to students.

Undertaking meetings with a diverse group of students broadened SAG members’ understanding of the spectrum of mental health conditions and wellbeing issues that many face during their time at university. Students said it allowed reflection on both their own mental health (*“*[it’s] *therapeutic to be around people who understand the struggles”* (R13)) and of others (*“I became more aware of the different mental and well-being issues* [in] *different student groups”* (R17)). One of the group reported the adoption of mental health interventions as a result of involvement in the SAG that improved their mood and focus, along with satisfaction in their studies.

Many students reported a positive impact in developing specific skills such as team working and public speaking, but also in feeling valued and in working on an important cause (*“it feels amazing to be doing something I really believe in”* (R10)). Participation provided insight into collaborative cross site research and the many challenges it poses. The SAG reported a significant increase in their understanding of the importance of incorporating students’ experiences and motives into the design of the interventions, and the impact of qualitative research methodologies. The intervention development was highlighted by many as particularly informative (*“It has made me think about my own career and what research I would like to carry out in the future”* (R1)).

Students were also asked to discuss negative aspects of participation, but none were reported.

#### Role

The SAG see their role both at the backstage and frontstage of Nurture-U, in the practical application of the ideas being proposed by the research team and in reflecting on how strategies may be received by the student population. Individuals’ backgrounds influenced their participation in the project: the SAG used their own experiences to inform ways the project could be implemented to have the most effective impact.

Aspects reported to make the student advisory role clear included the initial meetings and introductions, written information sent out prior to meetings, and the summaries following. One student said the name “Advisory Group” specified that students were providing advice on the project. One student was unsure how to define the role due to the renumeration, they were not sure if they could consider it employment. There were reports of challenges in keeping pace with project progress during peak academic activity.

#### Power sharing

Factors that made everyone feel part of a team included regular and clear communication, researchers’ attitudes and communication style, and being able to see where the advice had been taken on board. However, some students said that it was not always clear when advice or suggestions had been implemented, and that more training on the subject matter may increase equality. One student said that the website needed to emphasise the involvement of the student group.

Students reported *“always being listened to”* (R14) and that *“no opinion is incorrect”* (R4). The use of doodle polls to schedule the meetings was identified as a way of showing respect for schedules. One student specifically appreciated that uncertainties in the project were communicated clearly. When asked how things could be improved, several students highlighted the breakout rooms in Teams meetings as *“the weakest point”* (R3); that group sizes were too large to allow detailed discussion, some members not contributing, and discussion points occasionally being unclear. Others felt that there should be more in-person meetings, or a suggestion of separation between undergraduate and postgraduates. Several students asked for audio or video formats of the information provided, and one suggested a Nurture-U handbook. Students differed in opinions on whether information should be more concise: shorter guidance would be more accessible, but the granular detail would be lost.

Students reported feeling unable to participate in group discussions, especially if more confident members of the group dominate the conversation or where they felt distracted by their academic work. There were recommendations to structure the meetings around more individual methods of feeding back, such as Padlets, as it allows people to voice different opinions or opinions at a later date. More opportunity to interact with other students, for example in team-building exercises or an increase in in-person meetings, was suggested as beneficial in making collaborating as a team will become more seamless.

#### Diversity

Different genders, ethnicities, academic levels and backgrounds all incite different life challenges to mental health and well-being. Many students highlighted the lack of representation in the SAG of those who do not identify as female. Two students said there needs to be more students who are postgraduates or part-time, and one reported a lack of visible input from disabled people. One student said that there were many international students which skewed the conversations often towards their experiences. Students suggested more targeted advertising would be beneficial to improve diversity, to specific student societies and networks for example.

### Impact on the research team

The 6 researchers who had worked most closely with the SAG fed back their viewpoints through the questionnaire.

#### Overall impact

Researchers reported personal value from the SAG, improving their understanding of generational nuances. Typical comments were that students not only provided insight but also energy and practical ideas that have been crucial in shaping the project (*“such positive and constructive feedback, really helpful for the project”* R6). This was specifically the case in the development of the Toolkit and therapies, along with marketing and social media (specifics in Table 1). Students’ advice on language and communication methods was deemed particularly useful. The only negative impact reported was the time taken for student advisory input into data collection and analysis, which could risk delay to research timelines.

#### Role

Researchers viewed the role of the SAG as ensuring the project is inclusive and relevant. Researchers felt that the continued multi-faceted engagement of several SAG members reflected their understanding of the role. However, two researchers felt more transparency was needed in how exactly the SAG input steers the direction of the project (*“I think they understand we value student input but potentially may not be aware exactly how this is used or where it has been used in the research”* R4).

#### Power sharing

Researchers admitted difficulty in implementing all the SAG feedback, especially if there were differing opinions in the group. Researchers would then decide using their knowledge of the wider research context (e.g. *“we felt that within the context of the app, the likely people using it, and the type of problems it was aiming to address, that this didn’t feel like feedback we needed to implement”* R2). There was some reflection that constraints of the project, such as the structure of the original grant application and budgetary factors, led to final operational decisions being researcher rather than student led.

Researchers also suggested different methods of collecting student input, for example an online whiteboard where students can anonymously post, with more encouragement to contribute between meetings. Another suggestion to increase dialogue was to start a newsletter, especially as the different research workstreams start and there are results to share. Regular feedback was highlighted to increase positive relationships.

#### Diversity

Researchers were concerned that SAG membership was majority Exeter students, meaning a lack of perspective from the other participating universities. This was partly a consideration of how the engagement budget was split between sites. Additionally, the need to increase the ratio of those who dowq not identify as female was highlighted.

## Discussion

Critical reflection of the role of the SAG in the preliminary stages of this large-scale, national university mental health research project has identified successes, and, more importantly, areas for improvement. This evaluation has allowed identification of learning points not only for the project as it progresses, but also for other groups aiming to engage students in research projects or designing services. The impact of the SAG permeates every stage of Nurture-U: defining concepts through naming and branding, co-designing and refining apps and therapeutic tools that will be tested as the project progresses, co-creating a successful recruitment campaign, and disseminating relevant information in social media. This differs from many examples of public involvement in research, which often takes place in just one area or stage of projects [1]. The PEQG framework was useful in highlighting areas to improve: SAG members and research staff were consistent in reporting positively the respect and accessibility in the group, but highlighted ways to improve representativeness, transparency in communication, and sustainability going forward.

The SAG reported excitement in seeing a large-scale research project that aims to improve student wellbeing by seeking the experience from the current student on campus. However, the extent to whether these views are heard in the final research decision-making needs to be examined. The National Institute of Health Research’s distinctions between consultation, collaboration, and user-controlled research summarise the range of public involvement [11]. ‘Consultation’ involves collection of feedback and making changes as appropriate, ‘collaboration’ involves advisors making equitable key decisions, while ‘user-controlled’ research involves the public taking the lead. Nurture-U aims to be a collaborative project with many decisions shared, and in many areas this was the case—for example in naming and branding the project and designing the wellbeing toolkit. However, it is clear from this reflection exercise that at points students were consultants, providing advice on previously designed ideas. For example, the Toolkit software and therapy apps had been chosen in advance for student comment. The primary reason for this is that the team are working with strict methodologies and timelines which were set out in the funding application. Criticisms of power-sharing in research argue that these barriers are common, and hence it is often researchers who make the final decisions even when there is extensive public input [14].

However, this evaluation process has been a positive step in aiding the Nurture-U team to continue to strive for more equitable collaboration with the student group as the project progresses into the data collection, analysis, and dissemination stages. The co-authorship of this paper and the student-led social media campaigns demonstrate a willingness on both sides for joint ownership of these processes. The hope is that this will allow for continuity and sustainability of the project findings, as discussed in the PEQG framework [11]. There is also much to be learnt from the literature on student-staff collaboration within higher education more widely, which has been shown to increase student responsibility for learning and also engage staff to achieve objectives which would not have been possible alone [15]. However, this literature also highlights the need for both parties to take on new perspectives: staff to understand the effect of historical power imbalances on current relationships, and students to understand the university as a whole institution, in order for true collaboration to be possible [16, 17]. Harrison argues that engagement needs to have affective, cognitive, and behavioural elements in order to lead to true collaboration [18]. Only 15% of the whole SAG provided feedback for this paper, which suggests the majority of students engaging in the project may be missing one of these elements. One way to increase this may be to draw on the diverse experiences of the advisers that led them to join the project, and encourage reflection of the project within these experiences, instead of just asking for feedback.

SAG members and researches indicate that another way to encourage deeper collaboration would be to change the methods of communication. Suggestions for alternative ways for students to feedback on aspects of the project, such as providing more individual methods (e.g. Padlets), engaging with smaller groups, and having more in-person meetings will be implemented. A lack of clarity in how student feedback has been used was raised, which will be rectified with improved transparency of decision making processes [19]. This could be in short video summaries of the senior team meetings, or monthly newsletters. As the project progresses, and students are involved in analysis and dissemination of the data, the hope is that their roles and involvement will be more concrete. For example, the baseline survey results have been shared, which the SAG found exciting as they are a tangible results of their marketing and advertising.

Russell et al. [11] describe ‘norms of bureaucracy’ which results in a certain type of individual being the most likely to participate in research activities. There is a clear lack of diversity in the high proportion of female members of the SAG, although it should be noted that this is typical both for those who are interested in studying mental health, and in those who experience poor mental health [20, 21]. More work needs to be done in increasing ethnic diversity. Recruiting to public involvement groups is often challenging; increasing diversity will require additional effort to engage with student groups or organisations who infrequently participate in wellbeing activities [22]. We can do this by forming links with university societies and increasing our social media contacts to encourage a wider range of group members. Using these methods to encourage participation from the other Universities in the study may also increase the diversity of the SAG. There is also work to be done reducing the stigma of mental health, which may be especially important in recruiting more male members [8]. Other studies of including young people in development of mental health services have cited different methods, such as anonymous forums, or ‘think aloud’ activities, as ways to get those engaged who may otherwise not wish to take part [23].

The strengths of this study lie in its analysis of student engagement in research at an early stage of a large-scale national project, allowing for learning points to be taken forward not only in other projects but within Nurture-U as it progresses. The co-authorship of this paper is also important in ensuring its relevance and applicability. A limitation is the retrospective nature of this evaluation, in asking for feedback after tasks were completed. Ongoing reflection on engagement activities may have encouraged a higher proportion of students who have been involved in the SAG to provide feedback.

## Conclusions

Holmes et al. [24] report that the success criteria for public involvement for research include changes to research based on public feedback; creation of inclusive practices and environments that are valued; personal and/or career progression, including increased knowledge and skill for all involved; and maximum diversity of public contributors. Assessing against these criteria, we can consider the Nurture-U SAG successful, but this opportunity for formal reflection on the student involvement processes has been important in planning for growth and improvement as the project progresses.

## Data Availability

Anonymised data is available from the corresponding author on reasonable request.
